# Effects of quantum statistics of phonons on the thermal conductivity of silicon and germanium nanoribbons

**DOI:** 10.1186/1556-276X-8-7

**Published:** 2013-01-03

**Authors:** Yuriy A Kosevich, Alexander V Savin, Andrés Cantarero

**Affiliations:** 1 Semenov Institute of Chemical Physics, Russian Academy of Sciences, Moscow, 119991, Russia; 2 Materials Science Institute, University of Valencia, PO Box 22085, Valencia, 46071, Spain

**Keywords:** Thermal conductivity, Molecular dynamics simulation, Nanoribbon, Silicon, Germanium, Isotopic effect

## Abstract

We present molecular dynamics simulation of phonon thermal conductivity of semiconductor nanoribbons with an account for phonon quantum statistics. In our semiquantum molecular dynamics simulation, dynamics of the system is described with the use of classical Newtonian equations of motion where the effect of phonon quantum statistics is introduced through random Langevin-like forces with a specific power spectral density (color noise). The color noise describes interaction of the molecular system with the thermostat. The thermal transport of silicon and germanium nanoribbons with atomically smooth (perfect) and rough (porous) edges are studied. We show that the existence of rough (porous) edges and the quantum statistics of phonon change drastically the low-temperature thermal conductivity of the nanoribbon in comparison with that of the perfect nanoribbon with atomically smooth edges and classical phonon dynamics and statistics. The rough-edge phonon scattering and weak anharmonicity of the considered lattice produce a weakly pronounced maximum of thermal conductivity of the nanoribbon at low temperature.

## Background

It has been recently shown [1] that silicon and germanium nanowires can give a figure of merit of over 2 at 800 K due to strong reduction of phonon thermal conductivity in nanowires as compared with their equivalent bulk material, i.e., the reduction is caused not only by the alloy disorder, but also by the decrease of the phonon mean free path by reduced-dimensional effects. The effect of temperature on the thermal conductivity of silicon and germanium may be quite different since the Debye temperature of silicon almost doubles that of germanium. The purpose of the present work is to analyze quantum statistic effects on thermal phonon conductivity in silicon and germanium nanoribbons with the use of the novel *semiquantum* molecular dynamics simulation [2].

Molecular dynamics is a method of numerical modeling of molecular systems based on classical Newtonian mechanics. It does not allow for the description of pure quantum effects such as the freezing out of high-frequency oscillations at low temperatures and the related decrease to zero of heat capacity for *T*→0. On the other hand, because of its complexity, a pure quantum-mechanical description does not allow, in general, the detailed modeling of the dynamics of many-body systems. To overcome these obstacles, different semiclassical methods, which allow to take into account quantum effects, have been proposed [3-9].

The most convenient method for numerical modeling is to use the Langevin equations with color-noise random forces [5,7]. In this approximation, the dynamics of the system is described with the use of classical Newtonian equations of motion while the quantum effects are introduced through random Langevin-like forces with a specific power spectral density (the color noise), which describes the interaction of the molecular system with the thermostat. Here, we apply such semiquantum approach to the simulation of heat transport in low-dimensional nanostructures such as semiconductor nanoribbons with atomically smooth (perfect) and porous (rough) edges. Our previous analytical studies [10] and molecular dynamics simulations [11] have revealed the dramatic decrease of phonon thermal conductivity in quasi-one-dimensional nanostructures with rough (porous) surface and edge layers.

## Methods

In the semiquantum molecular dynamics approach, the dynamics of the system is described with the use of the classical Newtonian equations of motion while the effects of phonon quantum statistics are introduced through random Langevin-like forces with a specific power spectral density (the color noise). If the random forces are delta-correlated in a time domain, this corresponds to the white noise with a flat power spectral density. This situation corresponds to high-enough temperatures, when *k*_*B*_*T *is larger than the quantum of the highest phonon frequency mode in the system, $k_{B}T\gg \hbar \omega_{\text{max}}$. However, for low-enough temperature, $k_{B}T\ll \hbar \omega_{\text{max}}$, the stochastic dynamics of the system should be described with the use of random Langevin-like forces with a non-flat power spectral density, which corresponds to the system with color noise. For the generation of color noise with the power spectrum, consistent with the quantum fluctuation-dissipation theorem, we use the method which was developed in [2]. The semiquantum molecular dynamics approach has allowed us to model the transition in the rough-edge nanoribbons from the thermal insulator-like behavior at high temperature, when the thermal conductivity decreases with the conductor length (see [11]), to the ballistic conductor-like behavior at low temperature, when the thermal conductivity increases with the conductor length. Here, we apply the semiquantum molecular dynamics approach for the modeling of temperature dependence of thermal phonon conductivity in silicon and germanium nanoribbons with rough edges. We show that the presence of rough edges significantly decreases the room-temperature thermal conductivity of the nanoribbon and results in the weakly pronounced maximum of thermal conductivity at low temperatures. The latter property is closely related with the absence of (or weak) anharmonicity of the lattice potential and correspondingly weak anharmonic (Umklapp) scattering. In our semiquantum molecular dynamics approach, we make use neither of the quantum corrections to classically predicted thermal conductivity, e.g., discussed in [12], nor of the values of Umklapp or surface roughness-induced scattering rates, calculated independently from molecular dynamics simulation, e.g., discussed in [13,14]. To diminish the contact (interface) boundary resistance between the nanoribbon and heat reservoirs, e.g., discussed in [15], we model the nanoribbon with relatively long parts, immersed in semiquantum heat baths (see also [2]).

## Results and discussion

We consider a system which consists of *K* parallel atomic chains in one plane [11]. To model the diamond-like lattice, we assume that each atom has four nearest neighbors. In this connection, we would like to mention that the considered model cannot be applied directly to the predicted [16-19] and recently grown [20,21] two-dimensional lattice with graphene-like structure, made from Si or Ge atoms, the silicene. Our main goal is to provide semiquantum modeling of the heat transport and effective ‘isotopic effect’ on phonon heat transport in low-dimensional structures made from Si or Ge atoms, arranged in lattices, which reflect the symmetry of corresponding bulk materials. Since the lattice structure (the number of nearest neighbors) of the considered quasi-two-dimensional nanoribbons reflects the bulk one, our model can also be applied to the quasi-three-dimensional nanowires with bulk-like structure. The isotopic effect on phonon heat transport can be used for the understanding and prediction of the trends in the changes of thermal conductivity in low-dimensional nanostructures caused by the essential change in ion masses accompanied by less strong change in inter-ion force constants.

The Hamiltonian of the system describes the kinetic energy and harmonic interparticle interaction potentials. The characteristic energy of the nearest-neighbor interaction energy *E*_0_ can be related with the energy of the LO phonon mode in the semiconductor, which is approximately 15 THz in Si and approximately 9 THz in Ge. The ratio of these maximal frequencies is close to the ratio of the Debye temperatures, *T*_*D *_= 645 K in Si and *T*_*D *_= 374 K in Ge, and to the ratio of the inverse square root of Si and Ge atomic masses, which reflect the approximate isotopic effect in phonon properties of Si and Ge lattices when the materials can be described approximately with the same force constants and different atomic masses (see [22]). The particle mass (*M*) and lattice constant (*a*) are determined by the mass and characteristic period of the corresponding bulk semiconductor material, *a *= 5.43 Å and *a *= 5.658 Å for Si and Ge, respectively.

**Figure 1:**
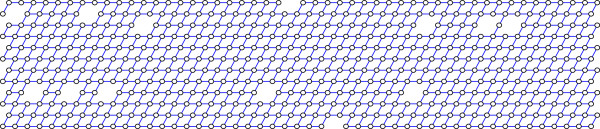
Nanoribbon with porous edges cut from two-dimensional diamond-like lattice where each atom has four nearest neighbors.

We consider a ribbon which consists of *K *= 18 atomic chains. To model the roughness of the ribbon edges, we delete with probability (porosity) *p *= 1−* d* some atoms from *K*_1_ chains adjacent to each ribbon edge. Here, *K*_1_ is a width of the rough edges, and *d*, 0 ≤* d *≤ 1, is a fraction of the deleted atoms in the edge atomic chains. In our simulations, we take *K*_1_ = 4 and *d *= 0.80. In Figure 1, we show an example of the nanoribbon with porous edges, cut from the two-dimensional diamond-like lattice in which each atom has four nearest neighbors.

**Figure 2:**
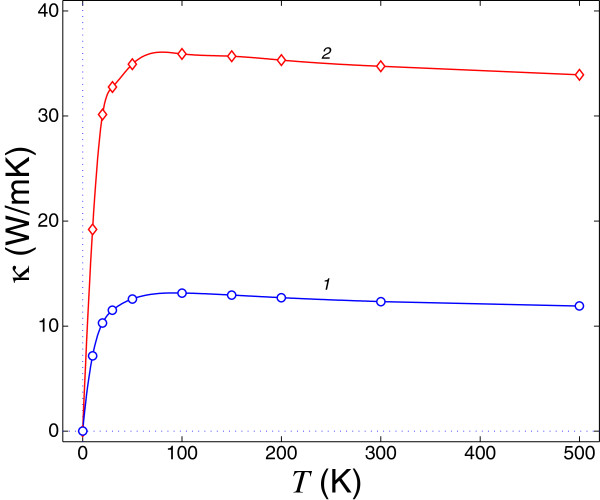
**Thermal conductivity *κ *of rough-edge nanoribbon versus temperature for ribbon length of *N *= 500 unit cells.** Thermal conductivity *κ *of rough-edge nanoribbon (ribbon width *K *= 18 atomic chains, rough edges widths *K*_1_ = 4 atomic chains, porosity of rough edges *p *= 0.20) versus temperature *T* for ribbon length of *N *= 500 unit cells of the two-dimensional diamond-like lattice of Ge (blue circles, line 1) or Si (red diamonds, line 2) atoms.

We computed the thermal conductivity *κ*(*NT*) for the nanoribbons with the length of *N *= 500 unit cells. In Figure 2, we plot the results of the semiquantum molecular dynamics simulation of thermal conductivity of Si and Ge nanoribbons. As one can see in this figure, the thermal conductivities of both Si and Ge nanoribbons have a weakly pronounced maximum at low temperatures, *T*_*max *_= 85 K for Si and *T*_*max *_= 91 K for Ge. This property of thermal conductivity temperature dependence is a consequence of rough-edge scattering as the main phonon scattering mechanism at elevated temperatures and the absence of (or weak) anharmonicity of the lattice potential and correspondingly the absence of (or weak) anharmonic (Umklapp) scattering. The latter causes a clear peak in the thermal conductivity versus temperature both in finite bulk crystals of pure silicon [23] and in low-dimensional nanoribbons [2]. The values of thermal conductivities of the Si and Ge nanoribbons for *T *>* T*_*max*_ approximately reproduce an isotopic effect because $\kappa \propto v_{\text{ph}}\propto 1/\sqrt{M}$, where *v*_*ph*_ is the group velocity of acoustic phonons (see also [22]). The weakly pronounced maximum of the thermal conductivity, at approximately 150 K, was recently observed in Si nanowires in [1]. We want to emphasize in this connection that thermal conductivities of the nanoribbons with the same widths, interparticle potentials, and perfect edges diverge in the limit of *N*→*∞* for all temperatures (see [2]). On the other hand, the obtained suppression of thermal conductivity in the rough-edge nanoribbons for the used value of surface porosity *p *= 0.20 is not so strong as that for the Si nanowires with rough surfaces which were studied recently in [24] (compare Figures 1 and 2 in this work with Figures one and three in [24]).

## Conclusions

Semiquantum molecular dynamics simulations with random Langevin-like forces with a specific power spectral density show that quantum statistics of phonons and porosity of edge layers dramatically change the thermal conductivity of Si and Ge nanoribbons at low and room temperatures in comparison with that of the nanoribbons with perfect edges and classical phonon dynamics and statistics. Phonon scattering by the rough edges and weak anharmonicity of the considered lattice produce weakly pronounced maximum of the thermal conductivity of the nanoribbon at low temperature. The approximate isotopic effect is manifested in the $\kappa \propto v_{\text{ph}}\propto 1/\sqrt{M}$ scaling of phonon thermal conductivities of the rough-edge nanoribbons with harmonic lattices at elevated temperature. This effect can be used for the prediction of the trends in the changes of phonon thermal conductivity in low-dimensional nanostructures, which was caused by the essential change in atomic masses accompanied by a less strong change in inter-atomic force constants.

## Competing interests

The authors declare that they have no competing interests.

## Authors’ contributions

This work was finished through the collaboration of all authors. YAK proposed the model for the lattice and isotopic effect. AVS has been working on the MD simulation. YAK and AC have participated in the interpretation of results and in revising the manuscript. All authors read and approved the final manuscript.
